# New isoform-specific monoclonal antibodies reveal different sub-cellular localisations for talin1 and talin2

**DOI:** 10.1016/j.ejcb.2011.12.003

**Published:** 2012-03

**Authors:** Uta Praekelt, Petra M. Kopp, Kerstin Rehm, Stefan Linder, Neil Bate, Bipin Patel, Emmanuel Debrand, Ana Maria Manso, Robert S. Ross, Franceso Conti, Ming-Zhi Zhang, Raymond C. Harris, Roy Zent, David R. Critchley, Susan J. Monkley

**Affiliations:** a Department of Biochemistry, University of Leicester, Lancaster Road, Leicester LE1 9HN, UK; b University Medical Center Eppendorf, 20246 Hamburg, Germany; c Department of Medicine, University of California at San Diego, School of Medicine, La Jolla, CA, USA; d VA Healthcare San Diego, CA 92161, USA; e Dubowitz Neuromuscular Centre, Institute of Child Health, University College London, London WC1N 1EH, UK; f Division of Nephrology, Department of Medicine, Vanderbilt University Medical Center, North Nashville, TN 37232, USA; g Department of Medicine, Veterans Affairs Hospital, Nashville, TN 37212, USA

**Keywords:** Integrin, Talin, Cytoskeleton, Focal adhesions, Fibrillar adhesions, Podosomes, Monoclonal antibody

## Abstract

Talins are adaptor proteins that connect the integrin family of cell adhesion receptors to cytoskeletal actin. Vertebrates express two closely related talins encoded by separate genes, and while it is well established that talin1 plays a key role in cell adhesion and spreading, little is known about the role of talin2. To facilitate such studies, we report the characterisation of 4 new isoform-specific talin mouse monoclonal antibodies that work in Western blotting, immuno-precipitation, immuno-fluorescence and immuno-histochemistry. Using these antibodies, we show that talin1 and talin2 do not form heterodimers, and that they are differentially localised within the cell. Talin1 was concentrated in peripheral focal adhesions while talin2 was observed in both focal and fibrillar adhesions, and knock-down of talin2 compromised fibronectin fibrillogenesis. Although differentiated human macrophages express both isoforms, only talin1 showed discrete staining and was localised to the ring structure of podosomes. However, siRNA-mediated knock-down of macrophage talin2 led to a significant reduction in podosomal matrix degradation. We have also used the antibodies to localise each isoform in tissue sections using both cryostat and paraffin-embedded material. In skeletal muscle talin2 was localised to both myotendinous junctions and costameres while talin1 was restricted to the former structure. In contrast, both isoforms co-localised in kidney with staining of the glomerulus, and the tubular epithelial and interstitial cells of the cortex and medulla. We anticipate that these antibodies will form a valuable resource for future studies on the function of the two major talin isoforms.

## Introduction

Cell adhesion to the extracellular matrix (ECM) plays a key role in the migration, proliferation and differentiation of animal cells, and their organisation into tissues and organs during embryonic development. Several types of cell–ECM junctions have been characterised in cultured cells (Dubash et al., 2009) including focal complexes formed at the leading edge of migratory cells, which in turn mature into larger more elongated focal adhesions (FA). Fibrillar adhesions (FB) are found in the central area of the cell and are associated with the fibronectin fibrillogenesis (Cukierman et al., 2001), while podosomes and invadopodia are more specialised adhesion structures that are only found in certain cell types (Block et al., 2008). All the above cell–ECM junctions share the same general architecture and composition, i.e. the extracellular domains of the integrin family of α/β hetero-dimeric trans-membrane proteins are bound to ECM proteins, while the short cytoplasmic tails of the integrin β-subunits are linked to the actin cytoskeleton via a variety of adaptor proteins (Brakebusch and Fassler, 2003; Legate and Fassler, 2009). One such adaptor is talin (∼270 kDa, ∼2540 amino acids) which binds both β-integrin tails and F-actin (Critchley, 2009), and also modulates the affinity of integrin for ligands (Anthis and Campbell, 2011; Shattil et al., 2010). Talin consists of an N-terminal head containing an atypical FERM domain (Elliott et al., 2010; Goult et al., 2010) that binds β-integrin tails (Anthis et al., 2009; Calderwood et al., 1999; Wegener et al., 2007) coupled to an elongated flexible rod with a second integrin binding site (Gingras et al., 2009; Moes et al., 2007), at least two actin binding sites (Gingras et al., 2008; Hemmings et al., 1996), and multiple binding sites for the cytoskeletal protein vinculin (Gingras et al., 2005). Much of talin in the cell is thought to exist in an autoinhibited cytoplasmic form due to intramolecular interactions between the head and rod (Goksoy et al., 2008; Goult et al., 2009), and both Rap1/RIAM (Han et al., 2006; Lee et al., 2009) and PIP2 (Goksoy et al., 2008; Martel et al., 2001) have been implicated in talin activation.

In vertebrates there are two talin genes, *Tln1* and *Tln2*, which encode very similar proteins (74% amino acid sequence identity) (Debrand et al., 2009; Monkley et al., 2001). *Tln2* appears to be the ancestral gene with *Tln1* arising by gene duplication early in the chordate lineage (Senetar and McCann, 2005). However, the role of the two major talin isoforms remains unclear. Knockout of *Tln1* is embryonic lethal at gastrulation (Monkley et al., 2000) while *Tln2* knockout mice are viable and fertile (Chen and Lo, 2005), although they have a mildly dystrophic phenotype that is more severe than that arising from muscle-specific knockout of *Tln*1 (Conti et al., 2008, 2009). Interestingly, talin2 has a much higher affinity for the cytoplasmic tail of β1D-integrin (Anthis et al., 2010), a splice variant that is localised with talin2 in the myotendinous junction of striated muscle. This suggests a model in which the tight binding of talin2 to β1D-integrin is designed to withstand the high forces exerted on the myotendinous junction in vivo. Loss of both talin1 and talin2 from muscle leads to severe defects in myogenesis and is perinatal lethal, indicating that the two isoforms have overlapping but non-redundant functions in muscle (Conti et al., 2009).

Further progress in understanding the function of talin1 and talin2 has been restricted by the lack of antibodies that are specific for each isoform. Many of the commonly used commercial antibodies, e.g. 8d4 and TD77 (Sigma) recognise both isoforms, while the only talin1-specific antibody (TA205) detects human but not mouse talin1 (Bolton et al., 1997). Here we characterise four new isoform-specific monoclonal antibodies (Mabs) that detect either talin1 or talin2 from a range of species, and that work in Western blotting, immuno-precipitation, immuno-fluorescence and immuno-histochemistry. We have used these antibodies to analyse the sub-cellular localisation and tissue distribution of both isoforms, and show for the first time that in NIH3T3 cells, smooth muscle cells, mouse embryo fibroblasts and macrophages, the sub-cellular localisation of these two very similar proteins is quite distinct.

## Materials and methods

### Ethical statement

All procedures on animals were approved by one of the following: (i) The Institutional Animal Care and Use Committee of Vanderbilt University according to the NIH Guide for the Care and Use of Laboratory Animals – protocol number M/04/219 from Vanderbilt Medical Center. (ii) The University College London local animal ethical committee review following guidelines set out by the 1986 UK Home Office Animal Procedures Act under the Home Office Licence PPL 70/7086.

### Monoclonal antibody generation

The recombinant talin immunogens were purified his-tagged polypeptides containing residues 489–911 of mouse talin1 and the corresponding region of mouse talin2 (residues 492–914), and residues 2300 to the C-terminus of mouse talin1 and the corresponding region of mouse talin2. Antibodies to talin1 and talin2 immunogens were raised in BALB/C mice at Harlan Laboratories, UK. Test bleeds were analysed by ELISA and Western blotting against recombinant proteins. Spleen cells were fused with NS0 cells, and hybridomas selected following screening by ELISA against recombinant protein. Positive hybridoma supernatants were immediately tested in Western blots against recombinant talin1 and talin2 polypeptides to check specificity, and selected hybridomas were sub-cloned twice The talin1- and talin2-specific antibodies described in this study are available from Cancer Research Technology (http://www.cancertechnology.com/tools/antibodies).

### Western blotting

Cells or tissues were lysed in Laemmli sample buffer (62.5 mM Tris–HCl pH 6.8, 20% glycerol, 2% SDS, 5% β mercaptoethanol), and extracted proteins were resolved by SDS-PAGE and blotted to PVDF membranes. Antibodies were used as follows: affinity purified 97H6, 68E7 and 121A used at 0.1 μg/ml and 93E12 hybridoma supernatant diluted 1/50. Other antibodies used were: anti-vinculin F9 (Santa Cruz), HRP-coupled anti-mouse and anti-rabbit were from GE Healthcare.

### Cell culture and transfection

NIH3T3 mouse fibroblasts, rat aortic smooth muscle cells and mouse embryo fibroblasts were grown in DMEM with 10% foetal calf serum at 37% in 10% CO_2_ and cultured on uncoated plastic dishes (VWR). Sub-confluent cells were trypsinised, washed in PBS and replated at a density of 4 × 10^4^ cells on uncoated glass coverslips in 24-well plates (Raymond A. Lamb). Cells were fixed after 2, 4, 6 or 24 h. Human primary macrophages were isolated from peripheral blood with a Ficoll gradient. The cells were left to adhere and cultivated for 7 days on plastic dishes in RPMI1640 with 20% human serum.

Mouse talin1 cDNAs and human talin2 cDNA were amplified by PCR, cloned into pEGFP-N1 (Clontech) and the constructs validated by sequencing. All DNA used for transfection experiments was purified using the Endofree MidiPrep Kit (Qiagen). Sub-confluent NIH3T3 cells were trypsinised, washed in PBS, and electroporated (6 × 10^6^ cells/ml) using a Microporator (Invitrogen) according to the manufacturer's instructions with 0.5 μg of plasmid DNA encoding GFP or GFP-talin1 or GFP-talin2 and/or 100 pmol of a Dharmacon ON-TARGET plus SMARTpool E-065877-00-0005 against mouse talin2.

### siRNA-induced talin2 knock-down in human macrophages

Human talin2-specific siRNAs were obtained from Thermo Scientific Dharmacon: siGENOME siRNA, Human TLN-2-03: 5′-GAUGUGCGAUCACCACUAU-3′ and Human TLN-2-02 5′-GGACGACCCUUCCAUGUAC-3′; control firefly luciferase siRNA#2 (Thermo Scientific) was used as negative control. Cells were transiently transfected using a MicroPorator MP-100 (PeqLab, Erlangen, Germany) combined with the 100 μl tips from NEON transfection system (Invitrogen, Darmstadt, Germany) using the following specifications: pulse voltage 1000 V, pulse width 40 ms, pulse number 2. Cells (1 × 10^5^) were seeded on coverslips, and 72 h later, either fixed and stained or whole cell lysates prepared for Western blotting.

### Matrix degradation assay

Porcine gelatin (Roth, Karlsruhe, Germany) was fluorescently labelled using NHS-rhodamine (Thermo Scientific, Rockford, IL) according to Chen et al. (1994). Coverslips were coated with labelled matrix solution, fixed in 0.5% glutaraldehyde (Roth, Karlsruhe, Germany) and washed with 70% ethanol and medium. Cells were seeded at a density of 8 × 10^4^ on coated coverslips, and fixed with 3.7% formaldehyde/PBS after 6 h. Quantification of NHS-rhodamine gelatin fluorescence intensities was performed using ImageJ software. For comparability, laser intensity was not changed between measurements. For each value, 3 × 20 cells were evaluated. Statistical analysis was performed with Graphpad Prism software. Differences between mean values were analysed using the Student's *t*-test.

### Immuno-fluorescence microscopy on cells in culture

Cells grown on glass coverslips were fixed and permeabilised in ice-cold methanol for exactly 1 min, and cells were then blocked with 2.5% normal goat serum and 2.5% normal mouse serum for 15 min before staining for F-actin with Alexa 647-phalloidin (1:200), anti-fibronectin (Sigma, 1:200) or HMα5.1 anti-α5 integrin (1:50; Santa Cruz) in 1% BSA in PBS + 2.4 mM EGTA, 2.4 mM MgCl_2_. Talin1 and talin2 were detected with Mabs 97H6 and 68E7 diluted 1:20 if supernatants were used, or 5 μg/ml if purified antibodies were used. Alexa-488 or Alexa-594 coupled secondary antibodies (Molecular Probes) were used at a dilution 1:200. Confocal laser scanning microscopy was conducted using either a Leica TCS SP5 system consisting of a Leica DMI-6000 CS inverted microscope or an Olympus FV1000 system with an inverted IX81 motorised microscope.

For quantification of talin1 or talin2 positive structures, epifluorescence microscopy images were analysed using ImageJ (NIH). After conversion to 8-bit images, the outlining tool was used to determine cell area, and a threshold of 20–250 pixels (equivalent to = 0.2–25 μm^2^) was set to measure the number and size of talin-positive adhesion complexes. This threshold was determined empirically beforehand to exclude signals derived from diffuse cytoplasmic staining for talin. For each experiment, 30 cells were analysed and the experiment was conducted in triplicate. A two-tailed unpaired Student's *t*-test was performed to test for significance.

### Immuno-staining of skeletal muscle cryosections

C57BL/6 or talin2-null mice were sacrificed, gastrocnemius muscles collected, immersed in OCT and snap-frozen in liquid nitrogen-cooled isopentane. For immunohistochemistry, 12 μm-thick sections were fixed in methanol at −20 °C for 5 min, incubated with 10% goat serum/PBS for 30 min and incubated with MOM kit Blocking Reagent (Vector Labs, CA) for 1 h. Sections were then washed three times in PBS, incubated with antibodies to talin1 (97H6) and talin2 (68E7) at 5 μg/ml for 1 h, followed by incubation with Alexa Fluor 594 for 1 h. Nuclei were counterstained with Hoechst 33342 (Invitrogen). Images were acquired with a Leica DM4000B microscope and processed using Metamorph and ImageJ software.

### Immuno-staining of formalin-fixed paraffin embedded sections of kidney

Mouse kidneys were perfused through a transcardial aortic cannula with 3.7% formaldehyde, 10 mM sodium *m*-periodate, 40 mM phosphate buffer and 1% acetic acid (which quenches endogenous biotin), then dehydrated and paraffin-embedded (Zhang et al., 2010). Sections were de-paraffinised, soaked in 100% ethanol, and then incubated for 20 min in 100% methanol containing 0.3% H_2_O_2_ to quench endogenous peroxidase activity. The sections were rehydrated, blocked with PBS containing 10% normal goat serum for 1 h at room temperature and then incubated with either isotype control or primary antibody diluted in PBS containing 1% normal goat serum overnight at 4 °C. Affinity purified 97H6, 68E7, and 121A were used at a concentration of 2 μg/ml, and 93E12 was used at a dilution of 1:10. Primary antibodies were localised using Vectastain ABC Elite (Vector, Burlingame, CA) with diaminobenzidine (DAB) as chromogen, followed by a light counterstain with toluidine (Zhang et al., 2010).

### Immuno-precipitation

NIH3T3 cell lysates (700 μg total protein) were incubated with 3 μg primary antibody (97H6, 68E7, 121A) or IgG control and rotated overnight at 4 °C. The following day, the solution was incubated with 50 μl protein G agarose beads (Roche) with rotation at 4 °C for 1 h. Beads were washed three times with lysis buffer (10 mM Tris–HCl, pH 8, 100 mM NaCl, 1% NP-40, 2 mM sodium orthovanadate, protease inhibitor cocktail (Roche)) and resuspended in 50 μl 2× Laemmli sample buffer. Samples were analysed by Western blotting using the appropriate antibodies (affinity purified 97H6 and 68E7, Vinculin (Sigma clone hVIN-1)), β1 integrin (BD Transduction clone 18/CD29).

## Results

**Supplementary Fig. S1:**
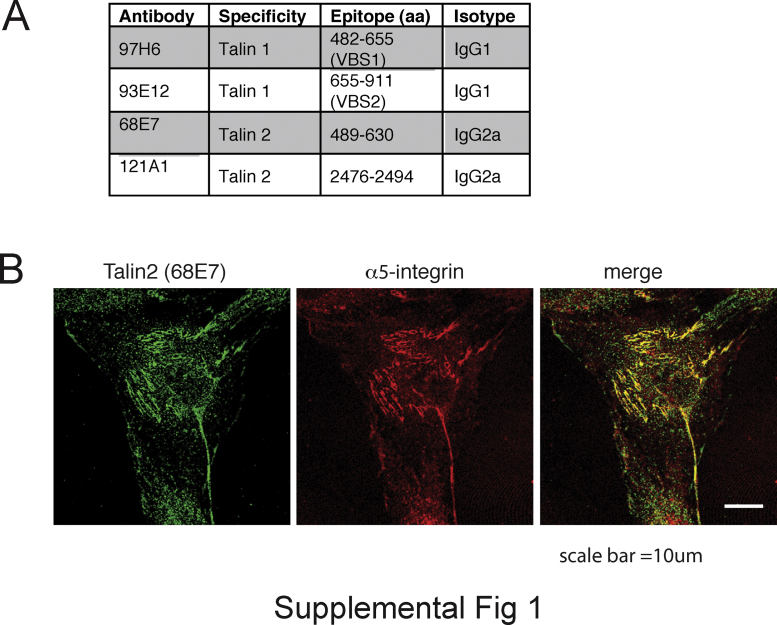
(A) Table summarising the characteristics of the various Mabs described in this study. (B) Confocal microscopy of NIH3T3 cells cultured on glass coverslips for 24 h and stained with antibodies to α5 integrin (red) and talin2 (68E7; green) as indicated. The merge of two images demonstrates significant overlap between talin2 and α5 integrin. Bar = 10 μm.

**Fig. 1:**
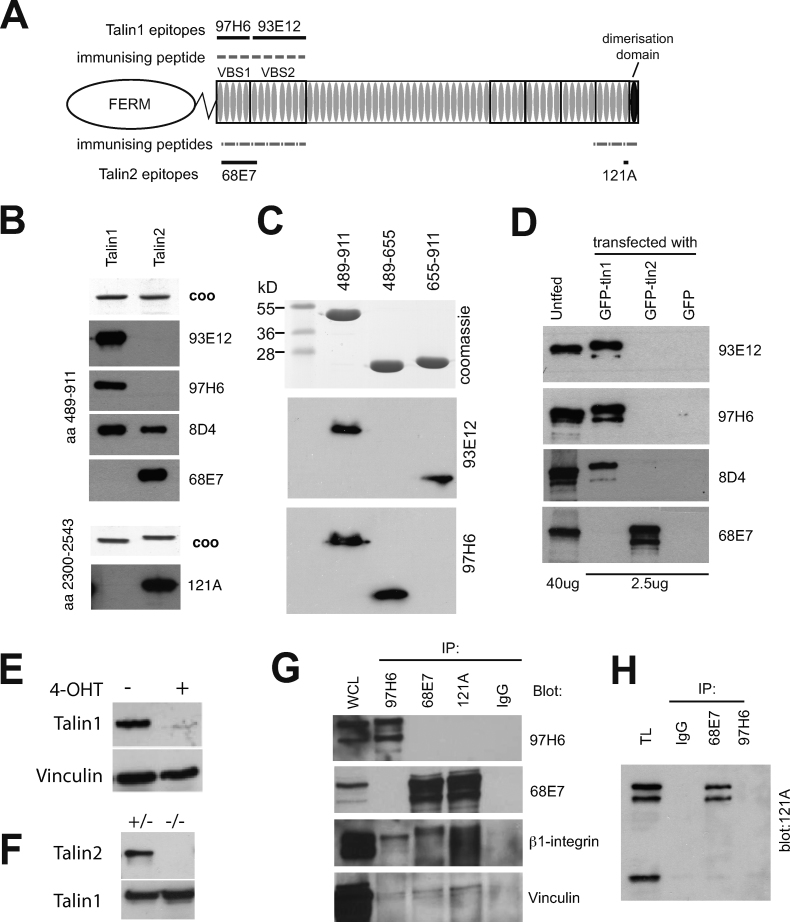
Characterisation of isoform-specific talin monoclonal antibodies. (A) Domain structure of talin. The N-terminal talin head, which is comprised of an atypical FERM domain, is linked to the talin rod by an unstructured region (zig-zag) containing a calpain-II cleavage site. The talin rod contains 61 α-helices (grey ovals) organised into a series of amphipathic α-helical bundles, and terminates in a single helix responsible for talin dimerisation. Boxes indicate established domain boundaries. The location of the recombinant polypeptides used to generate Mabs and the position of their epitopes is shown either above (talin1) or below (talin2) the diagram. (B) Purified recombinant talin rod polypeptides (50 ng) spanning residues 489–911 (upper panel) or 2300–2542 (lower panel) were resolved by SDS-PAGE and either stained with Coomassie blue (coo) or analysed by Western blotting using talin Mabs 97H6, 93E12, 68E7, 121A as well as the commercial talin Mab 8d4 (Sigma). (C) The epitopes recognised by Mab 93E12 and 97H6 were further defined using recombinant talin1 rod polypeptides containing either residues 482–655 or residues 656–911. Mab 97H6 recognises the former while 93E12 recognises the latter. (D) Western blots of whole cell lysates from untransfected mouse embryo fibroblasts (40 μg protein) or lysates from cells expressing either GFP-talin1, GFP-talin2 or GFP alone (2.5 μg protein) probed with the talin Mabs indicated. (E) Mouse embryo fibroblasts homozygous for a conditional *Tln1* allele and carrying a tamoxifen-inducible Cre recombinase were treated with 100 nM 4-hydoxy tamoxifen (4-OHT) dissolved in ethanol (+) or ethanol alone (−). After 72 h, cells were replated, and after a further 24 h analysed for talin1 expression by Western blotting using the 97H6 Mab. Vinculin was used as a loading control. (F) Heterozygous *Tln2* knockout mice (+/−) were crossed, and 12.5dpc (+/−) or (−/−) embryos analysed for expression of talin1 (97H6 Mab) or talin2 (68E7 Mab) by Western blotting. (G) Lysates from NIH3T3 were immunoprecipitated with either a control IgG, the talin1-specific Mab 97H6 or the talin2-specific Mabs 68E7 and 121A, and the immunoprecipitates and whole cell lysate (WCL) analysed by Western blotting using the talin1-specific Mab 97H6, the talin2-specific Mab 68E7, the β1-integrin Mab (BD Transduction) and a vinculin Mab (Sigma). (H) Tissue lysates (TL) from mouse kidney were immunoprecipitated with either a control IgG, the talin2-specific Mab 68E7 or the talin1-specific, Mab 97H6 and immunoprecipitates analysed by Western blotting using the talin2-specific Mab 121A. The ∼90 kDa protein detected by Mab 121A in kidney TL is a C-terminal talin2 polypeptide spanning residues 1608–2543 (Debrand et al., 2009) which is not recognised by the 68E7 Mab.

Monoclonal antibodies (Mabs) recognising either the N- or the C-terminal regions of the talin1 or talin2 rod domain (Fig. 1A) were generated by immunising mice with recombinant talin polypeptides as described in the “Materials and methods” section. Two Mabs (93E12 and 97H6) were identified that recognised the talin1 but not talin2 immunogen in Western blots (Fig. 1B). Both were immunoglobulin type IgG1 (Fig. S1A), and using overlapping polypeptides, we showed that they recognised different epitopes (Fig. 1C). 97H6 recognised residues 482–655, a 5-helix bundle at the start of the talin1 rod (Papagrigoriou et al., 2004) while 93E12 recognised residues 655–911 (Fig. 1A and C). We also generated Mab 68E7, a talin2-specific Mab against the N-terminal region of the talin2 rod (Fig. 1B), and epitope mapping showed that it recognised residues 489–655 (data not shown). A commonly used commercial talin Mab 8d4 also recognises the N-terminal region of talin1 in Westerns (Fig. 1B), but it also detected the equivalent region of talin2, albeit to a lesser degree. A second talin2-specific Mab, 121A, was obtained from immunisations with a C-terminal talin2 polypeptide (Fig. 1A and B). The epitope was mapped by peptide display using the *E. coli* protein EspA (Crepin et al., 2005) to the 14 amino acids (KAAFGKADDDDVVV) spanning residues 2476–2494 (data not shown). Both talin2-specific antibodies were of the IgG2b isotype (Fig. S1A).

In order to confirm the ability of these antibodies to detect full-length talins, we tested them against lysate from cells expressing either GFP-talin1, GFP-talin2 or GFP alone by Western blotting (Fig. 1D). Both 97H6 and 93E12 detected only GFP-talin1 (or the endogenous talin1 in untransfected cells), but not GFP-talin2. Similarly the talin2 Mabs 68E7 (Fig. 1D) and 121A (not shown) recognised only GFP-talin2 plus the endogenous talin2 in untransfected cells. The commercial Mab 8D4 detected primarily GFP-talin1 (Fig. 1D) although upon longer exposure it also detected GFP-talin2 (not shown). The specificity of the talin1 antibodies was further demonstrated using mouse embryo fibroblasts derived from mice carrying conditional *Tln1* and *Cre/ER* alleles. Activation of Cre recombinase with 4-hydroxy tamoxifen (4-OHT) inactivates the *Tln1* gene and resulted in near complete loss of the talin1 signal as detected using the 97H6 Mab (Fig. 1E). Because the *Tln2* gene is far more complex, it has not been possible to use a similar approach to generate *Tln2* null cells (Debrand et al., 2009). We have therefore deleted the entire coding region of the *Tln2* gene (Debrand et al., in preparation), and Western blots of *Tln2*^(−/−)^ embryos show a complete loss of talin2 protein as detected using the 68E7 Mab (Fig. 1F).

The talin1 97H6 Mab and the two talin2 Mabs 68E7 and 121A were also able to specifically immuno-precipitate their respective antigens from NIH3T3 cells along with the talin-binding partners β1-integrin and vinculin (Fig. 1G). Interestingly, immuno-precipitation of talin1 with Mab 97H6 did not lead to coimmuno-precipitation of talin2 either from NIH3T3 cells (Fig. 1G) or mouse tissues lysates (Fig. 1H). Similarly, immuno-precipitation of talin2 with Mabs 68E7 or 121A did not lead to co-immuno-precipitation of talin1 demonstrating for the first time that the two isoforms do not form heterodimers.

### Use of isoform-specific antibodies to study the sub-cellular localisation of talin1 and talin2

**Fig. 2:**
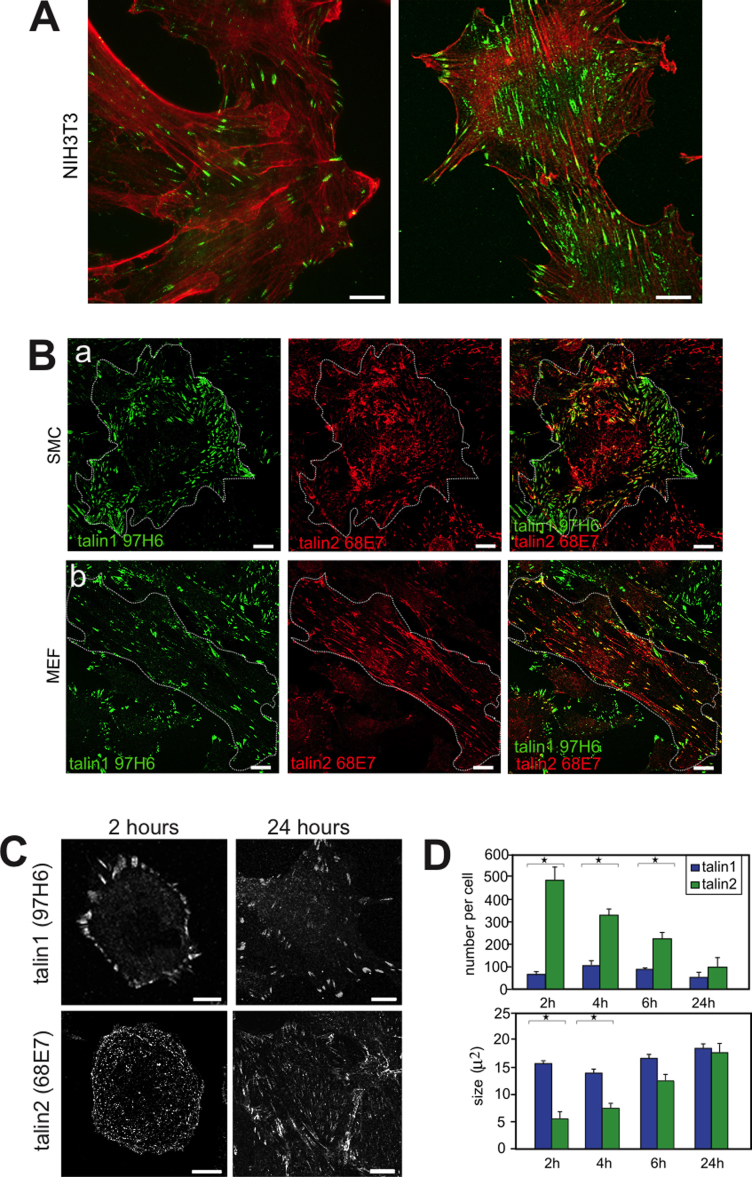
Sub-cellular localisation and dynamics of talin1 and talin2 in various cell types. NIH3T3 cells, smooth muscle cells (SMC) and mouse embryo fibroblasts (MEF) were grown on glass coverslips. (A) Confocal microscopy images of cells stained separately with Mabs to either talin1 (97H6) or talin2 (68E7). (B) Confocal microscopy images of cells co-stained with both talin1 and talin2 Mabs (97H6 and 68E7) followed by isotype-specific secondary antibodies (talin1 97H6 IgG1, talin2 68E7 IgG2b). Talin1 (green); Talin2 (red). (C) NIH3T3 cells stained for talin1 or talin2 either 2 h or 24 h after replating onto glass coverslips. Scale bars = 10 μm. (D) Quantification of number and size of talin1 or talin2 positive structures from (C) 2, 4, 6 or 24 h after replating. All results are expressed as mean ± SEM where *N* = 30 per time point. *P*-values for number of talin1 versus talin2 structures per cell: 2 h: 0.0006; 4 h: 0.0001; 6 h: 0.0005; 24 h: 0.089. Size of talin1 versus talin2 structures per cell: 2 h: 0.0001; 4 h: 0.0004; 6 h: 0.074; 24 h: 0.4; *P values of < 0.05. *Note*: The size of talin2 positive FB at later time points is underestimated due to the “thresholding” method (see the “Materials and methods” section) used to exclude cytoplasmic staining of talin which overlaps talin2 staining in central FB. (For interpretation of the references to colour in this figure legend, the reader is referred to the web version of the article.)

To establish whether the isoform-specific Mabs were suitable for sub-cellular localisation studies, we initially used NIH3T3 cells, which express both talin isoforms. Both the talin1-specific 97H6 and the talin2-specific 68E7 Mabs worked most effectively on methanol-fixed cells rather than paraformaldehyde/TritonX100 or formaldehyde/acetone-treated cells (data not shown). Interestingly, while talin1 was primarily localised in FA found at the cell periphery, talin2 was found in both FA and elongated structures throughout the body of the cell (Fig. 2A) suggesting that the two isoforms have different functions. We therefore compared the localisation of talin1 and talin2 in several other cell types using isotype-specific secondary antibodies to identify both talin1 and talin2 in the same cell. The picture that emerges is essentially the same as in NIH3T3 cells, and is illustrated here with images from rat aortic smooth muscle cells and mouse embryo fibroblasts (Fig. 2B). Thus, talin1 was localised to FA whereas talin2 was found in both FA and structures in the centre of the cell that aligned in the same direction as actin stress fibres (not shown), and resemble fibrillar adhesions (FB).

**Fig. 3:**
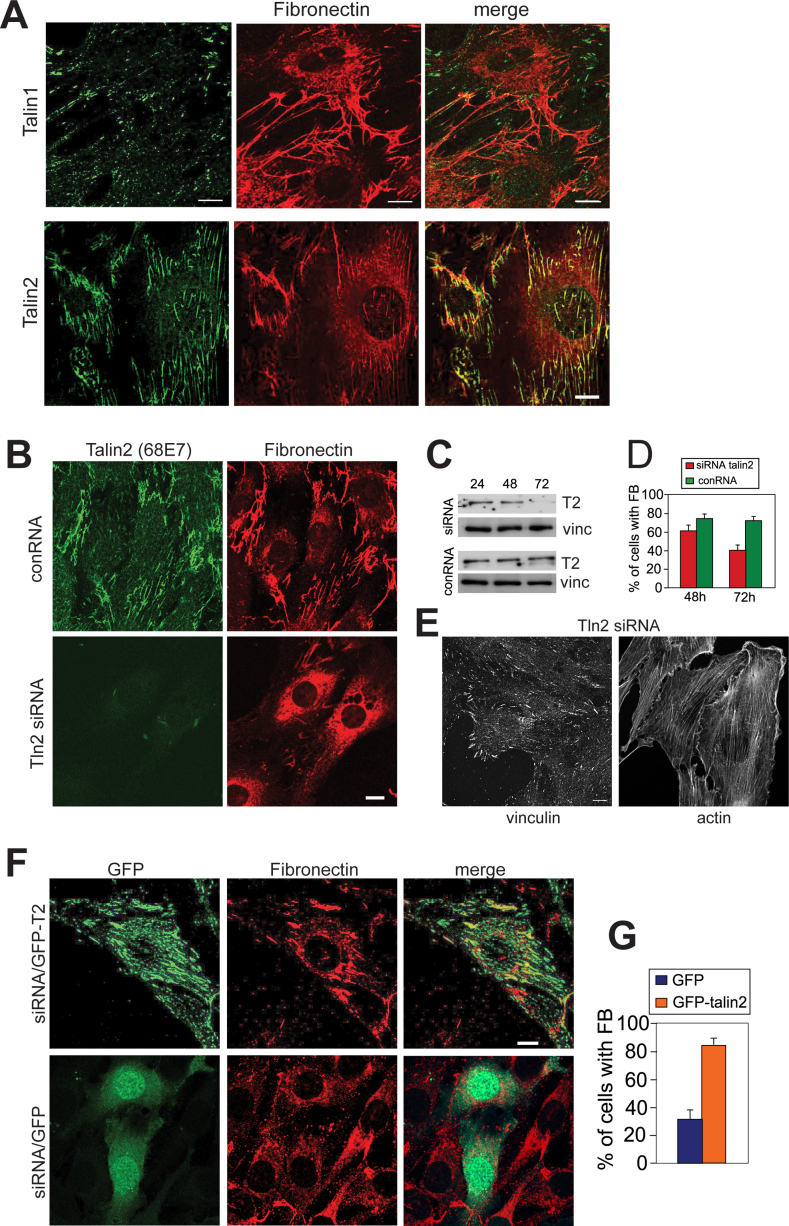
Talin2 localises to fibrillar adhesions and talin2 knock-down leads to a decrease in fibronectin fibrils. (A) Confocal microscopy of NIH3T3 cells cultured on glass coverslips for 24 h and stained for talin1 (97H6; top row) or talin2 (68E7; bottom row) and fibronectin. (B) NIH3T3 cells were transfected with 100 pmol of either a SMARTpool siRNA against mouse talin2 or a control siRNA, and 72 h post transfection were fixed and stained for talin2 (68E7) and fibronectin. (C) Cells transfected with the talin2 siRNA or control siRNA were analysed by Western blotting for talin2 (68E7) or vinculin 24, 48 and 72 h after transfection. (D) Percentage of cells with FB 48 h or 72 h after transfection with the talin2 siRNA or control siRNA. (E) NIH3T3 cells transfected with the talin2 siRNA and stained for either vinculin or F-actin (phalloidin) 72 h after transfection. (F) Confocal images of NIH3T3 cells transfected with either human GFP-talin2 or GFP together with the mouse talin2 siRNA, followed 72 h later by staining for fibronectin. (G) Quantitation of FB in NIH3T3 cells 72 h after transfection with human GFP-talin2 or GFP along with the mouse talin2 siRNA. Error bars are ±SEM. *N* = 30. Scale bars = 10 μm.

When NIH3T3 cells were stained at different times after replating, talin2 was initially found in a large number of small dot- and rod-shaped structures spread evenly across the cell (Fig. 2C). Over time, these structures appeared to fuse, and their number decreased while their size increased (Fig. 2C and D). In contrast, the talin1-positive FA changed very little in number or size over this period (Fig. 2C and D). Thus, the two talin isoforms show both different dynamics as well as sub-cellular localisations in NIH3T3 cells, with talin1-positive FA forming within the first hour after replating, while formation and maturation of the talin2-containing structures occurred over a much longer time frame. To establish if the talin2 containing structures were FB, NIH3T3 cells were co-stained with antibodies to fibronectin (Fig. 3A) and α5-integrin (Fig S1B). This showed that there was substantial co-localisation between talin2, fibronectin and α5-integrin particularly in the centre of the cell. Talin1 localisation on the other hand was quite distinct from that for fibronectin (Fig. 3A).

The localisation of talin2 predominantly to FB prompted us to explore its possible role in fibronectin fibrillogenesis, and we therefore performed talin2 siRNA knock-down experiments in NIH3T3 cells. Transfection of cells with a talin2 Dharmacon siRNA SMART pool led to a substantial reduction in talin2 levels as judged by immunofluorescence (Fig. 3B) and Western blotting (Fig. 3C) at the 72 h time-point, further demonstrating the specificity of the talin2 antibodies. However, talin1 expression was unaffected (data not shown), and there appeared to be no change in FA or stress fibre formation (Fig. 3E), or in cell spreading (Fig. 3B) compared to cells transfected with the control siRNA. In contrast, the number of cells with fibronectin fibrils was reduced 72 h post-transfection from 75% to 40% (Fig. 3B and D). Instead, fibronectin staining was localised around the nucleus (Fig. 3B). Importantly, re-expression of GFP-tagged human talin2 in cells depleted of mouse talin2 restored FB formation (Fig. 3F and G). In summary, the results demonstrate that talin1 and talin2 localise to different structures in three different cell types, and that in NIH3T3 cells, talin2 is required for efficient fibronectin fibrillogenesis.

### Talin1 but not talin2 is localised in podosomes

**Fig. 4:**
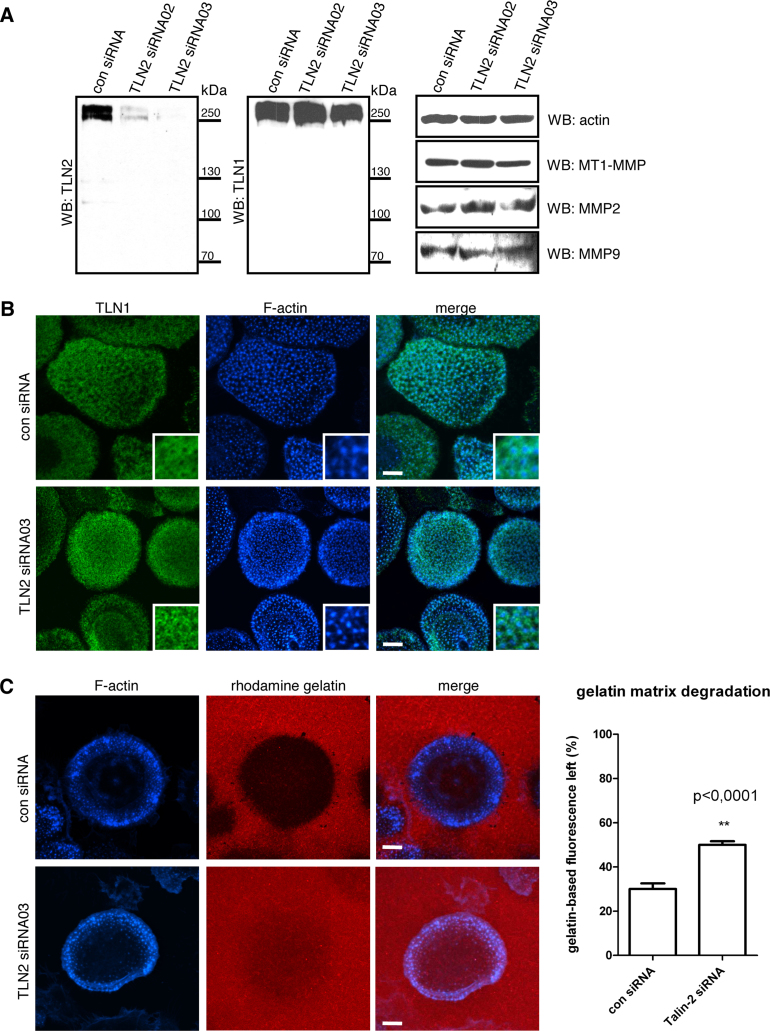
Knock-down of talin2 leads to a reduction of podosomal matrix degradation in primary human macrophages. (A) Western blots of macrophage lysates 72 h after transfection with the talin2 siRNAs indicated. Blots were probed for; left – talin2 (Mab 121A1), middle – talin1 (Mab 97H6), right – β-actin loading control and the matrix metalloproteinases (MMP) indicated. MMP2 and MMP9 rabbit polyclonal antibodies were obtained from Santa Cruz and the anti MT1-MMP antibody was from Millipore. (B) Primary human macrophages transfected with a control luciferase siRNA (upper panels) or a talin2 siRNA (lower panels) for 72 h, were seeded on coverslips, stained for talin1 (green) or F-actin (blue) and imaged by confocal laser scanning microscopy. Note localisation of talin1 to podosome rings surrounding F-actin-rich cores. (C) Knockdown of talin2 reduces podosome-dependent gelatin matrix degradation. Human macrophages transfected with a talin2 siRNA or control luciferase siRNA for 72 h were seeded on a rhodamine-labelled gelatin matrix for 6 h, and then fixed and stained for F-actin (blue). Dark areas indicate the region of matrix degradation. The degree of gelatin matrix degradation was quantified (±SD) by measuring the primary fluorescence under each of 20 cells using confocal laser scanning microscopy. The experiments were conducted in triplicate. The fluorescence intensity of the undegraded labelled matrix was set at 100%. Asterisks indicate a *P* value < 0.0001. Magnification bar = 10 μm. (For interpretation of the references to colour in this figure legend, the reader is referred to the web version of the article.)

It has been reported that talin1 is the only talin isoform expressed in cells of the haematopoetic lineage such as B cells, platelets and dendritic cells (Lammermann et al., 2008; Manevich-Mendelson et al., 2010; Nieswandt et al., 2007; Petrich et al., 2007). However, our microarray data suggest that talin2 expression is upregulated as monocytes differentiate into macrophages (S. Linder et al., unpublished data), and Western blotting using the isoform-specific antibodies confirmed that both talin1 and talin2 are expressed in differentiated human peripheral blood macrophages (Fig. 4A). It is also noteworthy that the antibodies did not cross-react with any other macrophage proteins further demonstrating their specificity.

One of the major adhesion structures in macrophages is the podosome, which can be distinguished from other adhesion structures by its characteristic two-part architecture consisting of an F-actin rich core surrounded by a ring structure containing many of the proteins typically found in FA (Linder and Kopp, 2005). We therefore stained differentiated macrophages with the isoform-specific talin antibodies. Interestingly, we found that while talin1 was localised to the outer ring structure (Fig. 4B), talin2 was not present in podosomes, a finding confirmed using the two different talin2-specific Mabs (data not shown) that recognise different regions of the protein (Fig. 1A). Indeed, we saw no specific talin2 staining in macrophages, and both the diffuse and the punctate staining observed on the ventral surface in a few cells appeared to be non-specific as it remained after siRNA talin2 knock-down.

To establish whether talin2 has any functional relevance in primary macrophages, we knocked talin2 protein levels down using an siRNA approach. Western blotting confirmed that cells treated for 72 h with two different talin2 specific siRNAs showed a specific reduction of talin2, but not talin1 compared to cells treated with the luciferase control siRNA (Fig. 4A). This result was confirmed by immunofluorescence staining for talin1 which remained localised in the podosome outer ring structure (Fig. 4B). Moreover, the number of podosomes per cell was unchanged in talin2 knock-down cells (not shown). Talin2 knock-down and control cells were subsequently seeded on a fluorescently labelled gelatin matrix, and after 6 h, the degree of matrix degradation was evaluated by fluorescence intensity measurements of the area covered by cells. For control cells, the matrix-associated fluorescence intensity was reduced to 30.01 ± 2.54%, while for talin2 knock-down cells, the value was only reduced to 49.98 ± 1.66% (Fig. 4C). Importantly, the talin2 siRNAs did not affect the levels of the matrix-metalloproteinases MT1-MMP, MMP2 and MMP9 (Fig. 4A). These findings indicate that although depletion of talin2 has no apparent effect on the localisation of talin1 in podosomes, or on the number of podosomes per cell, it does reduce the matrix-degrading capacity of macrophages without affecting the expression of MT1-MMP, MMP2 and MMP9.

### Tissue distribution of talin1 and talin2

**Fig. 5:**
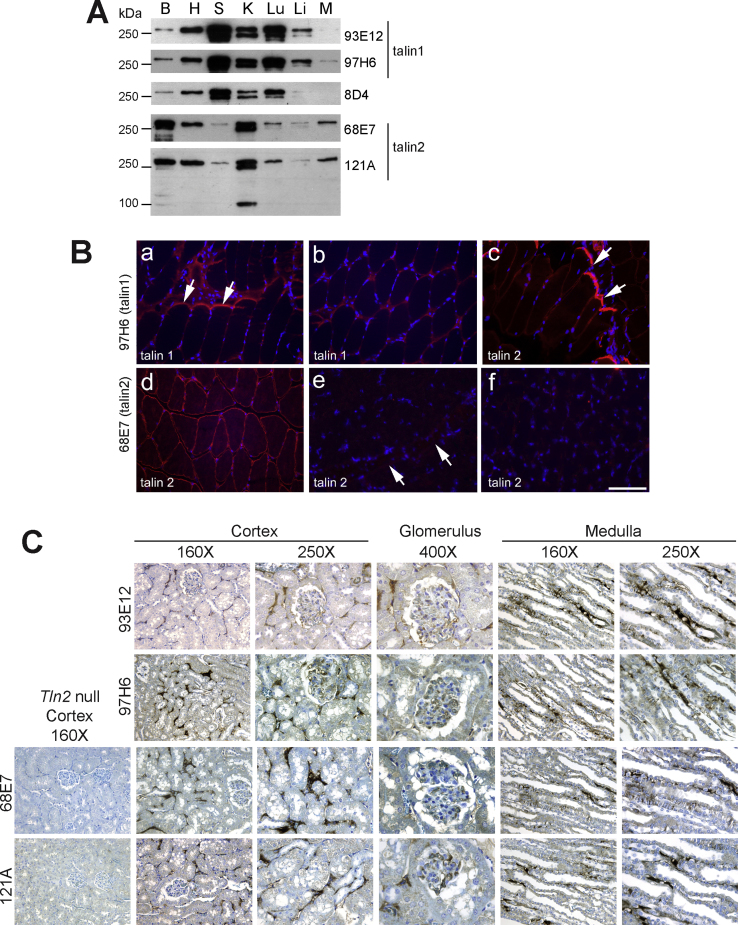
Expression of talin1 and talin2 in various mouse tissues and their immuno-localisation in muscle and kidney. (A) Western blot of whole tissue lysates (40 μg protein) probed with the talin1- or talin2-specific antibodies shown. Tissues: B, brain; H, heart; S, spleen; K, kidney; Lu, lung; Li, liver; M, skeletal muscle. (B) Cryo-sections of gastrocnemius muscle were immunostained with Mab 97H6 directed against talin1 (a and b) and Mab 68E7 against talin2 (c–f). Talin1 was mostly localised to the myotendinous junction (MTJ) (arrows in a), with weaker staining of costameres (b), while talin2 staining was strong at both MTJ (arrows in c) and costameres (d). In talin2-null muscle, no staining was detected at the MTJ (arrows in e) or at costameres (f). Scale bar = 50 μm. (C) Immuno-staining of paraffin-embedded sections of wild type and talin2 null mouse kidney with antibodies to talin1 (93E12 and 97H6) and talin2 (68E7 and 121A). Staining is present in the glomerulus, tubules and interstitium in the cortex, and the tubules and interstitium in the medulla. There was no staining for talin2 in kidneys from *Tln2*-null mice.

Previous data indicates that talin1 and talin2 mRNAs are differentially expressed in both mouse and human tissues (Debrand et al., 2009; Monkley et al., 2001). To get an indication of the relative protein abundance, we used the isoform-specific talin antibodies to carry out Western blotting on a range of mouse tissues (Fig. 5A). We found that talin1 appeared to be the predominant isoform in spleen, liver and lung while talin2 was the predominant isoform in brain and skeletal muscle. In heart and kidney, both appeared to be expressed about equally. As Mab 121A is directed to the C-terminal region of talin2, it detects both full-length (270 kDa) talin2 and the short 90 kDa kidney isoform that is expressed from an internal promoter (Debrand et al., 2009). The commercial antibody 8D4 gave an expression profile more like that of talin1 consistent with the conclusion that it has a higher affinity for talin1.

To establish whether these antibodies were able to detect the two major talin isoforms in tissues, we initially stained cryostat sections of skeletal muscle where talin2 is the predominant isoform (Fig. 5A). In agreement with previous studies using rabbit polyclonal antibodies (Conti et al., 2009), talin2 showed strong staining in both the myotendinous junctions and costameres of mouse gastrocnemius muscle (Fig. 5B c–e), and importantly, there was no staining for talin2 in muscle from talin2 knockout mice (Fig. 5B e and f). In contrast, talin1 staining was mostly localised to the myotendinous junction with very little staining apparent in costameres (Fig. 5B a and b). Given the relative low abundance of talin1 in muscle, the lack of costamere staining could be a sensitivity issue. Nevertheless, the results clearly show that talin1 in muscle is predominantly localised at the myotendinous junction.

We also sought to establish whether the antibodies worked on paraffin-embedded formalin fixed tissue by staining sections of kidney which contains substantial amounts of both talin isoforms (Fig. 5A). Sections stained positively for both talin1 (Mabs 93E12 and 97H6) and talin2 (Mabs 68E7 and 121A), and each showed similar staining patterns (Fig. 5C). Staining was present in the glomerulus and also in tubular epithelial and interstitial cells of both the cortex and the medulla. No staining was observed in kidney sections stained with the appropriate isotype controls (data not shown), and no staining was observed in talin2 null kidneys using either the 68E7 or 121A Mabs (Fig. 5C).

## Discussion

Here, we describe four new Mabs specific for talin1 or talin2 that are suitable for Western blotting, immuno-precipitation, immuno-fluorescence and immuno-histochemistry on both cryo-sections and formalin-fixed paraffin-embedded material. The isoform specificity of the antibodies was confirmed by Western blotting of cells expressing GFP-tagged recombinant talins, conditional talin1 knockout cells and talin2 knockout embryos, by immuno-precipitation and by the loss of talin2 staining in muscle and kidney sections from knockout mice. We have also previously shown that talin1 staining is lost in frozen sections of developing embryoid bodies from talin1(−/−) ES cells, while talin2 staining was unaffected (Liu et al., 2011). While other isoform-specific antibodies for talin2 have been described (Senetar et al., 2007), these are the only Mabs reported that recognise mouse talins and that are suitable for a wide range of uses.

Using these antibodies, we report for the first time clear differences in the sub-cellular localisation, dynamics and functions of the two talins. In fibroblasts, talin1 was principally localised in FA while talin2 was in both FA and FB, and was required for fibronectin matrix assembly. Talin1 and talin2 also showed differential localisation within muscle whereas in kidney, they showed co-localisation. Unlike other cells of the haemopoetic lineage, differentiated macrophages expressed both talin isoforms although only talin1 was localised in podosomes. However, talin2 knock-down significantly reduced the ability of macrophages to degrade matrix pointing to a podosome-related function of talin2.

Previous studies suggest that *Tln2* is the ancestral gene (Senetar and McCann, 2005). It is a large gene (>400 kb) due to the large size of the introns, encodes several splice variants and contains a number of promoters (Debrand et al., 2009). Indeed, testis and kidney express much smaller variants of talin2 as a result of alternate promoter usage. *Tln1* on the other hand evolved more recently, and is a much smaller gene (∼30 kb) with a less complex gene structure, although the boundaries of the coding exons are totally conserved. Both genes are widely expressed, although Western blotting shows that the relative level of the major isoforms varies substantially between tissues. Whereas most cells appear to express both isoforms, cells of haemopoetic origin and endothelial cells only express talin1, and talin2 is not upregulated when talin1 is depleted from endothelial cells (Kopp et al., 2010). The talin2 promoter lies within a CpG island (Debrand et al., 2009) and in haemopoetic cells may be silenced by methylation.

Little is known about the biochemical differences between the two major talin isoforms. Talin1 is a dimer, and dimerisation is mediated by the C-terminal helix (Gingras et al., 2008) which has a very similar sequence in talin2, raising the possibility that the two isoforms might form heterodimers. However, using the isoform-specific antibodies, we show that immuno-precipitation of talin1 from NIH3T3 cells and mouse tissues does not bring down talin2 and vice versa, indicating that talins exist as homodimers. This is entirely consistent with immuno-localisation studies which show that the two isoforms are differentially distributed within the same cell. Talins binds to β-integrin cytoplasmic tails via their N-terminal FERM domains (Anthis et al., 2009, 2010) although there is also an integrin binding site in the talin rod (Gingras et al., 2009; Moes et al., 2007). Interestingly, the talin2 FERM domain has a slightly higher affinity for β-integrin tails than the talin1 FERM domain, and in particular, it binds to the muscle-specific β1D-integrin splice variant far more tightly than any other known interaction between β-integrin subunits and talin. Both a structural (Anthis et al., 2009) and thermodynamic (Anthis et al., 2010) explanation for this observation has recently been reported, and the tight binding is consistent with the localisation of β1D-integrin and talin2 to the MTJ where they are thought to be important in withstanding the forces exerted on the MTJ during muscle contraction (Conti et al., 2008, 2009).

Otherwise, there is no data on novel binding partners that might account for the different localisation of talin1 and talin2 in other cell types. The finding that talin1 rapidly localises to FA whereas recruitment of talin2 to FB is significantly slower suggests that they have distinct functions and also modes of regulation. Talin1 is negatively regulated by a head/rod interaction that masks the integrin binding site in the FERM domain (Goksoy et al., 2008; Goult et al., 2009), and PKC signalling, Rap1 and its effector RIAM have all been shown to be important in talin1 and hence integrin activation (Han et al., 2006; Lee et al., 2009). Whether this same pathway is involved in regulating talin2 has not been explored. However, despite differences in their dynamics, endogenous talin2 compensates for loss of talin1 in fibroblasts (Zhang et al., 2008), and the effects of talin1 knock-down in endothelial cells (which only express talin1) can be rescued by transfection of a talin2 cDNA (Kopp et al., 2010). In contrast, talin2 appears unable to compensate for loss of talin1 in early embryonic development (Monkley et al., 2000, 2011). Moreover, although talin1 and talin2 co-localise in embryoid bodies at the epithelial/basement membrane junction, talin1-null embryoid bodies are defective in integrin adhesion complex assembly and signalling, and elongation of the embryonic epithelium, despite the continued presence of talin2 (Liu et al., 2011). The underlying cause appears to be increased proteasomal destruction of epithelial β1 integrin in the absence of talin1. We show here that talin1 and talin2 also co-localise in kidney tubular epithelial cells, and it will be interesting to explore their relative roles in kidney development and function.

The talin2-positive elongated adhesions in the centre of the cell reported here appear to be FBs since the talin2 staining is coincident with that for fibronectin and α5-integrin. Interestingly, in the first publication on talin (Burridge and Connell, 1983), the authors noted talin staining associated with fibres in the cell centre partially co-localising with fibronectin, but they assigned the staining to stress fibres instead of FB which had yet to be described. The authors also reported talin staining as having a “doughnut-shaped appearance with a non-fluorescent core surrounded by a fluorescent ring”. Presumably these were podosomes or invadosomes. More recently, talin2 staining has been described as “long adhesions in the midbody of the cell” (Senetar et al., 2007). Here, we show that talin2 knock-down in NIH3T3 cells compromised FB but not FA assembly, and the cells mislocalised fibronectin despite the continued expression of talin1. This and the fact that the talin2 knock-down phenotype was rescued by expressing a human talin2 cDNA strongly suggests a unique role for talin2 in FB formation and fibronectin fibrillogenesis. The current model of FB formation envisages that α5-integrins and the adaptor protein tensin translocate out of FA along actin stress fibres in a process that involves ILK (Stanchi et al., 2009), Src-mediated tyrosine phosphorylation and myosin II activity (Zamir and Geiger, 2001). Our recent studies show that it is the tensin3 isoform that is enriched in FB while tensin2 is enriched in FA (Clark et al., 2010). However, knock-down of tensin3 did not block fibronectin matrix assembly in human fibroblasts unlike the effects of talin2 knock-down in NIH3T3 cells reported here. Early on in cell spreading, we observed a multitude of talin2-positive dot and rod shaped structures in the centre of the cell that appeared to fuse over time into larger fibrillar structures that partially co-localised with fibronectin. This suggests that talin2 (but not talin1) may play a role in activating α5β1 integrin in the centre of the cell, coupling it to the actomyosin contractile apparatus which is essential for both FB and fibronectin matrix assembly.

The finding that talin1, but not talin2, is localised in podosomes was unexpected. Talin1 localised to the outer ring of the podosome which contains integrins and associated proteins typical of FA, as well as regulatory proteins such as PI3K, Src, Pyk2/FAK (Linder and Aepfelbacher, 2003). However, it is puzzling that we were unable to detect any specific staining for talin2 in differentiated macrophages under the conditions tested. Nevertheless, the fact microarray data indicate that talin2 may be upregulated during monocyte to macrophage differentiation (S. Linder, unpublished data) suggests an important role of talin2 in macrophage physiology. Indeed, matrix degradation assays showed that knock-down of talin2 significantly impaired the matrix degrading ability of primary human macrophages without affecting expression of matrix metalloproteinases, and it will be interesting to establish whether macrophages isolated from talin2 knockout mice are functionally impaired. Germline deletion of the talin2 gene does not result in any obvious phenotype and the mice are viable and fertile (Chen and Lo, 2005; Debrand et al., 2009), although maintaining a colony of mice in which the complete *Tln2* gene has been deleted is challenging for reasons that are not yet understood (Debrand et al., unpublished data). Again, it will be interesting to investigate whether macrophage-dependent responses to pathogens are impaired in *Tln2* null mice. In conclusion, it is clear that talin1 and talin2 have distinct functions at least in some cell types, and the availability of the isoform-specific antibodies reported here provide a valuable tool for future studies.
