# Supplementary material for: New isoform-specific monoclonal antibodies reveal different sub-cellular localisations for talin1 and talin2
Source: Eur J Cell Biol. 2012 Mar;91(3):180–91. doi: 10.1016/j.ejcb.2011.12.003 (PMC3629562; doi:10.1016/j.ejcb.2011.12.003)
Supplement: Supplementary file 1 [file mmc1.doc]

Ms. Ref. No.: EJCB-D-11-00140
Title: New isoform-specific monoclonal antibodies reveal different sub-cellular localisations for talin1 and talin2
European Journal of Cell Biology

| Received | Aug 24, 2011 |
| --- | --- |
| Decision | Oct 18, 2011 |
| Re-Submission received | Dec 13, 2011 |
| Accepted | Dec 19, 2011 |

**Decision letter**

Dear Dr. Critchley,

Your manuscript has now been seen by two referees whose comments are enclosed. As you will see from their reports, they both are, in principle, in favour of publication. However, they also make suggestions for a number of improvements that must be incorporated into a revised version of your manuscript. In particular, the question of proper control experiments requires special attention. If you feel you can meet the objections raised and supplement the manuscript accordingly, I should be happy to receive such a revised manuscript.

Please submit a list of changes or a rebuttal against each point which is being raised when you submit the revised manuscript.

Yours sincerely,

Manfred Schliwa
Editor

p.p. Dagmar Gebauer
Editorial Office
European Journal of Cell Biology

Reviewers' comments:

Reviewer 1

In the article entitled "New isoform-specific monoclonal antibodies reveal different sub-cellular localisations for talin1 and talin2", Praekelt and coauthors report the generation of new monoclonal antibodies that can distinguish between murine talin1 and talin2. The show that these antibodies are suitable for Western blot, immunoprecipitation, immunocytochemistry and immunohistochemistry. In the process, they demonstrate that talin1 and talin2 do not heterodimerize, and that they have distinct subcellular localization patterns in cells that express both isoforms. Furthermore, they show that talin2 has specific functions in fibronectin fibrillogenesis and matrix degradation in macrophages. Therefore, in addition to introducing two specific functions for talin2, the authors contribute new tools that will prove invaluable in further establishing distinct properties of the two talin genes. However, characterization of these new tools could be a little more thorough, for example by using talin1- or talin2-null cells and lysates as negative controls, and talin2-null muscle as a negative control. It appears that only the kidney immunohistochemistry used talin2 tissue as a negative control, and while knockdown of talin2 shows the specificity of the talin2-specific antibodies in cell staining, a similar approach to showing talin1 antibody specificity was not shown. The inclusion of these experiments, and attention to the points below, should merit publication of this work in EJCB.

1.     Slices from Western blots show that these antibodies can discriminate between talin isoforms, but do not provide insight into the degree of cross-reactivity with other proteins. Whole Western blots should be shown in the supplement so the reader can see how clean these antibodies are.
2.     Connected to this point, talin2 knockdown still gave staining in macrophages with the anti-talin2 antibodies. How many macrophage proteins do these antibodies recognise?
3.     Talin2 is apparently required for macrophage-mediated matrix degradation. Does talin2 knockdown affect the expression of the necessary proteases? I'm concerned about the possibility of unfortunate off-target effects.


Reviewer 2
The authors developed a set of isoform selective monoclonal antibodies and
studied differential expression and subcellular localization of talin 1 and
talin 2 protein in cells and tissue. In the light of talin's important role in
the control of integrin-based cell adhesion, this manuscript provides a
valuable contribution towards a better understanding of talin isoform
function. The authors observe differential association of both proteins with
specialized adhesion structures and reveal selective involvement of talin 2 in
FB formation / fibronectin fibrillogenesis and its relevance to matrix
degradation by macrophages. This set of well-characterized isoform selective
monoclonal antibodies will provide very useful tools to the community.

The manuscript is recommended for publication with minor revision.


Minor points

(1) Fig. 2B; SMC: intensity blot is inverted; images should be turned 90°
counter clockwise;
MEF: position of cells is hard to discriminate (dense cells, no nuclear
staining), and line scan (intensity blot) appears to run laterally alongside
rather than across the cell(s).

(2) Quantitative determination of adhesions sites (Fig. 2D) is not described
sufficiently in MATERIALS AND METHODS or RESULTS. Furthermore, which criteria
were used to identify FBs; length/shape and /or distance from plasma membrane
(Fig. 2D/G)?

(3) Fig 2D/3D/3/G: Do error bars indicate standard deviation or SEM? Was
statistical significance tested? N: number of cells analyzed for time points?

(4) Fig. 2C NIH 3T3 cells are not in the same phase of spreading; for talin1 a
polarized cells was selected, while a round cell (early spreading, not yet
polarized) was chosen for talin2.
If talin2 localized predominantly to elongated fibrillar adhesions in NIH 3T3
as suggested (Fig, 2A and 3A), why didn't staining for talin2 reveal larger
size (length in microns) on the average as compared to talin1, at 24 hours
(Fig. 2D)?

(5) Fig. 4C and Results:  Reduction of fluorescence intensity after matrix
degradation is given as 70% and 50% in controls and TLN2 knock down,
respectively. Rhodamine gelatine signal appears to be untouched (0% reduction)
in the representative image (TLNs siRNA03).

(6) Results / macrophages and Fig. 5A: talin2 staining does not always appear
to be specific. Thus in supplements, a representation of full-length gels from
tissue extracts would be useful.

(7) Fig 5B; Biochemical data suggest a special function of talin2 in MTJ,
where talin1 is also present, and not costameres. Could absence of costamere
staining be a consequence of detection limits and low talin1 expression in
gastrocnemius?

(8) Fig. 5C: Kidney sections are stained positively for talin1 and talin2.
Further details are not discernible at the resolution available. In
particular, staining at the basolateral membrane of tubular epithelial cells
is not evident. Diffuse interstitial staining may arise from blood cells.

Further, kidney, liver and spleen usually contain high level of endogenous
biotin. How did the author block endogenous biotin in this study? Did they
perform isotype controls to measure the level of non-specific background
signal caused by primary antibodies?
---------------------------------------------------------------------------------------------------------------------------------------------------------

**Authors response letter**

**Reviewer 1**

However, characterization of these new tools could be a little more thorough, for example by using talin1- or talin2-null cells and lysates as negative controls, and talin2-null muscle as a negative control. It appears that only the kidney immunohistochemistry used talin2 tissue as a negative control, and while knockdown of talin2 shows the specificity of the talin2-specific antibodies in cell staining, a similar approach to showing talin1 antibody specificity was not shown. The inclusion of these experiments, and attention to the points below, should merit publication of this work in EJCB.

*Talin1:*

*We have shown elsewhere that the protein recognized by the talin1-specific monoclonals in Western blots is lost in talin1 conditional knockout mouse embryo fibroblasts (Monkley et al., (2011) Dev Biol. 349, 494-502 – see Fig 1B) and that talin1 staining is lost in talin1 null embryoid bodies (Liu et al., Mol Cell Biol. (2011) 31, 3366-3377 – Fig 3B).*

*However, for completeness, we now include Western blot data on Talin1 knockout cell (revised Fig 1E).*

*We have also previously shown that talin1 immuno-staining is lost in frozen sections of developing embryoid bodies derived from talin1(-/-) ES cells while talin2 staining was unaffected (Liu et al., Mol Cell BIol. (2011) 31, 3366-3377 – Fig 3A).*

*Talin2:*

*We have not published data showing that the protein recognized by the talin2-specific monoclonals is lost in talin2 knockout cells, and we now include this data in the revised Fig 1F. We also include data to show that talin2 staining in muscle is lost in talin2 knockout mice as requested by the reviewer (revised Figure 5B c-f).*

*We now include reference to these studies in the first part of the discussion on page 13.*

1.      Slices from Western blots show that these antibodies can discriminate between talin isoforms, but do not provide insight into the degree of cross-reactivity with other proteins. Whole Western blots should be shown in the supplement so the reader can see how clean these antibodies are.

*We do not observe cross-reactivity of either the talin1 or talin2 antibodies with other cellular proteins. To illustrate this point, whole blots for macrophages are shown in the revised Figure 4A.*

2.      Connected to this point, talin2 knockdown still gave staining in macrophages with

the anti-talin2 antibodies. How many macrophage proteins do these antibodies recognise?

*First, it is important to state that punctate and diffuse staining for talin2 was only seen in a small proportion of macrophages. Second, as for point 1 above - whole blots for macrophages clearly demonstrate that the antibodies do not cross-react with other cellular proteins as judged by Western blotting (revised Figure 4A).*

3.      Talin2 is apparently required for macrophage-mediated matrix degradation. Does talin2 knockdown affect the expression of the necessary proteases? I'm concerned about the possibility of unfortunate off-target effects.

*Data to show that MT1-MMP, MMP2 and MMP9 are not affected by siRNA-mediated talin2 knockdown are now included in revised Figure 4A.*

**Reviewer 2**

Minor points

(1) Fig. 2B; SMC: intensity blot is inverted; images should be turned 90°

counter clockwise; MEF: position of cells is hard to discriminate (dense cells, no nuclear staining), and line scan (intensity blot) appears to run laterally alongside rather than across the cell(s).

*We have removed the intensity plot from revised Fig 2 because we felt that it added little to the conclusion that talin1 is localized in focal adhesions while talin2 is in both focal and fibrillar-like adhesions. The position of cell boundaries is now shown in revised Figure 2B.*

(2) Quantitative determination of adhesions sites (Fig. 2D) is not described

sufficiently in MATERIALS AND METHODS or RESULTS.

*We have revised M&M on page 7 to address this point.*

Furthermore, which criteria were used to identify FBs; length/shape and /or distance from plasma membrane (Fig. 2D)?

*Fibrillar adhesions were initially identified using fibronectin and α5-integrin staining, and we show that talin2 staining co-localises with α5-integrin (Figure S1B) and with fibronectin (Figure 3A). Fibrillar adhesions were subsequently identified as talin2 positive structures with an elongated shape.*

(3) Fig 2D/3D/3/G: Do error bars indicate standard deviation or SEM? Was statistical significance tested? N: number of cells analyzed for time points?

*Error bars in Figure 2/3 refer to SEM and this is now made clear in the relevant Figure legends as is the value of N. The results of statistical significance tests are also now included in the legend to Fig 2.*

(4) Fig. 2C NIH 3T3 cells are not in the same phase of spreading; for talin1 a polarized cells was selected, while a round cell (early spreading, not yet polarized) was chosen for talin2.

*We have replaced the 2h talin1 panel in Fig2C with a more representative image.*

If talin2 localized predominantly to elongated fibrillar adhesions in NIH 3T3 as suggested (Fig, 2A and 3A), why didn't staining for talin2 reveal larger size (length in microns) on the average as compared to talin1, at 24 hours (Fig. 2D)?

*This is due to the fact that we used “thresholding” to quantify talin1 and talin2 positive adhesion complexes in order to exclude cytoplasmic staining of the auto-inhibited form of talin which overlaps staining of central fibrillar adhesions. As a result, we "lose" the signal from the larger FB. We have added a note to the Figure 2D legend to explain this. However, this does not affect the overall conclusion which is that the number of talin2 positive structures falls with time as they get larger, whereas the number of talin1 positive structures and their size stays relatively constant.*

(5) Fig. 4C and Results:  Reduction of fluorescence intensity after matrix degradation is given as 70% and 50% in controls and TLN2 knock down, respectively. Rhodamine gelatine signal appears to be untouched (0% reduction) in the representative image (TLNs siRNA03).

*Good point. As suggested, we have exchanged the original image for a more representative one.*

(6) Results / macrophages and Fig. 5A: talin2 staining does not always appear to be specific. Thus in supplements, a representation of full-length gels from tissue extracts would be useful.

*We have addressed these issues in response to reviewer 1. Thus, revised Fig 4A shows full-length Western blots for talin1 and talin2 antibodies in macrophage lysates, which clearly demonstrates no unexpected cross-reactivity with other cellular proteins. The revised Fig 5B shows no staining in skeletal muscle from talin2 knockout mice using the talin2 antibody*

(7) Fig 5B; Biochemical data suggest a special function of talin2 in MTJ, where talin1 is also present, and not costameres. Could absence of costamere staining be a consequence of detection limits and low talin1 expression in gastrocnemius?

*Published data (Conti et al., 2008; Senetar & McCann 2005; Monkley et al., 2001) and the Western blot data in Fig 5A show lower expression of talin1 than talin2 in skeletal muscle. Lack of talin1 staining in costameres could therefore be a sensitivity issue. Nevertheless, what is clear is that talin1 in muscle is predominantly in myotendinous junctions. We have briefly discussed this on page 13 of the revised manuscript.*

(8) Fig. 5C: Kidney sections are stained positively for talin1 and talin2. Further details are not discernible at the resolution available. In particular, staining at the basolateral membrane of tubular epithelial cells is not evident. Diffuse interstitial staining may arise from blood cells.

*We quite accept the point raised, and have revised the description of the results on page 13.*

Further, kidney, liver and spleen usually contain high level of endogenous biotin. How did the author block endogenous biotin in this study?

*We have provided more details of the methods used to block endogenous biotin and indeed peroxidase in the revised M&M on pages 7/8.*

Did they perform isotype controls to measure the level of non-specific background signal caused by primary antibodies?

*Sections were stained with isotype-matched controls in place of the primary antibody (M&M page 8) and we observed no staining under these conditions although the data is not shown (Results page 13).*
